# Exploring changes in open defecation prevalence in sub-Saharan Africa based on national level indices

**DOI:** 10.1186/1471-2458-13-527

**Published:** 2013-05-30

**Authors:** Deise I Galan, Seung-Sup Kim, Jay P Graham

**Affiliations:** 1 Department of Environmental and Occupational Health, School of Public Health and Health Services, The George Washington University, 2100 M St. NW, Suite 203M, Washington, DC 20037, USA; 2 Department of Healthcare Management, Korea University, Seoul, Republic of Korea; 3 Department of Global Health, School of Public Health and Health Services, The George Washington University, 2175 K St. NW, Suite 200, Washington, DC 20037, USA

**Keywords:** Open defecation, Sanitation, Sub-Saharan Africa, Sanitation policy, Economic development, Total sanitation

## Abstract

**Background:**

In sub-Saharan Africa, it is estimated that 215 million people continue to engage in open defecation. This practice facilitates the transmission of diarrheal diseases – one of the leading causes of mortality in children under 5 in sub-Saharan Africa. The main purpose of this study is to: estimate changes in open defecation prevalence between 2005 and 2010 across countries in sub-Saharan Africa; examine the association between national level indices and changes in open defecation prevalence; and assess how many countries can achieve ‘open defecation free status’ by 2015.

**Methods:**

After applying selection criteria, this study analyzed country-level data for 34 sub-Saharan African countries. Seven country-level indices were collected: 1) presence of a national sanitation policy; 2) budget line for sanitation; 3) budget allocated to sanitation; 4) annual per capita GDP; 5) GDP growth; 6) implementation of total sanitation approaches; and 7) per capita aid disbursement for water supply and sanitation. The relationships between these country-level indices and the change in open defecation from 2005 to 2010 were investigated using Wilcoxon Signed-Rank test and Spearman's rank correlation test.

**Results:**

Only 3 countries (i.e. Ethiopia, Angola and Sao Tome and Principe) decreased open defecation by 10% or more between 2005 and 2010. No significant associations were observed between the change in open defecation prevalence and all of national level indices except per capita aid disbursement. Per capita aid disbursement for water and sanitation was positively associated with a reduction in open defecation (p-value = 0.02) for a subset of 29 low-income countries from 2005 to 2010. Only one country in our analysis, Angola, is on track to end open defecation by 2015 based on their performance between 2000 and 2010.

**Conclusions:**

Most of the national level indices, including a country’s economic status, were not associated with the change in the open defecation prevalence. Based on current trends, the goal of ending open defecation in the majority of sub-Saharan African countries by 2015 will not be achieved. Our findings may be limited by the exploratory nature of this analysis, and future research is required to identify and characterize national level factors specific to reducing open defecation in sub-Saharan Africa.

## Background

The practice of open defecation (hereafter, OD) facilitates the transmission of pathogens that cause diarrheal diseases – the second leading contributor to the global burden of disease, as measured in disability-adjusted life years (DALYs) [1-4]. It is estimated that 1.7 billion cases of diarrhea occur every year, causing approximately 800,000 deaths among children under 5 years of age worldwide [5,6]. It is estimated that 1.1 billion people – 15% of the global population – still engage in OD [7]. The majority of OD practices, referred to in national surveys as defecating in fields, forests, bushes, bodies of water or other open spaces, take place in rural areas of low-income countries. Even though the proportion of people practicing open defecation in sub-Saharan African has decreased by 11% from 1990 to 2010, the absolute number of people practicing OD has actually increased by 33 million over the same time period, due to population growth [7]. In 2010, OD was practiced by 8% of the urban population and 35% of the rural population in sub-Saharan Africa [7].

Although OD is not directly mentioned in the United Nation’s Millennium Development Goals (MDGs), reductions in OD are known to be critical to achieving MDG Target 7C that aims to “halve, by 2015, the proportion of people without access to safe drinking water and basic sanitation” [8]. Despite some progress being made globally, the WHO/UNICEF Joint Monitoring Program (JMP) for Water Supply and Sanitation (the official United Nations group assigned to monitor progress towards the MDG Target 7C) states that “it is unlikely that the world will meet the MDG sanitation target by 2015” [7]. In 2010, more than 2.5 billion people still lacked access to improved sanitation, which is defined by the JMP as sanitation facilities that hygienically separates human excreta from human contact. Improved facilities include flush or pour-flush toilets (flushed into a piped sewer system, septic tank or pit latrine), ventilated improved pit latrines, pit latrines with a slab and composting toilets [7]. Unimproved sanitation includes flush or pour-flush toilets that do not flush into a piped sewer system, septic tank or pit latrine; pit latrines without a slab or open pits, bucket latrines, hanging toilets or hanging latrines, shared or public facilities and open defecation [7]. In order to meet the sanitation MDG, sub-Saharan Africa will need to have 64% of its population covered by improved sanitation facilities, and the current trend indicates it will achieve only 32%. Only 9 countries on the African continent are on track for such reduction, and only 2 of those 9 are in sub-Saharan Africa [7].

Ending OD is not just a matter of access to sanitation facilities: it also involves motivational drivers such as prestige, well-being, and situational goals [9]. There is increasing value placed on motivating people to end OD, as evidenced by the United Nation’s new Sanitation Drive 2015 advocacy campaign working to end OD—even if the resulting sanitation facility does not meet the standards of improved sanitation. Along these lines, new approaches are being implemented in an effort to reduce OD. The most promising approaches, referred to in this paper as total sanitation approaches, aim to empower communities as a whole to become ‘OD free’. In contrast to past approaches that focused on individual households, total sanitation approaches target communities as a whole. Furthermore, total sanitation approaches promote use of local sanitation options that are based on affordability and available resources and reduce the role of hardware subsides. This approach aims to raise awareness of the risks associated with OD and generates a collective sense of intolerance towards OD [10-12]. A number of questions remain regarding the effectiveness of this approach, especially in urban areas where communities may be less cohesive. There are also questions about the durability of the sanitation systems built as they are often inadequately constructed. Thus, many organizations are applying hybrid approaches that integrate more market-based methods that aim for both economic sustainability and the installation of better quality sanitation systems. Nevertheless, it has been suggested that total sanitation approaches can result in rapid, significant improvements, and holds promise for decreasing open defecation in sub-Saharan Africa [13].

It is critical to understand what factors influence the pace at which countries are able to reduce OD in order to develop effective strategies to improving sanitation and reducing diarrhea morbidity and mortality caused by the lack of sanitation. Furthermore, there is a need for more realistic targets for global campaigns that will likely be put forth following the MDGs that end in 2015. In order to determine what factors may contribute to the reduction in OD, we analyzed country-level data for factors considered to be important to reducing OD for all sub-Saharan countries from 2005 to 2010. To the best of our knowledge, this is one of the first exploratory analyses of what national factors may contribute to reducing OD in sub-Saharan Africa. The goals of this study are to: 1) estimate changes in OD prevalence between 2005 and 2010 across countries in sub-Saharan Africa; 2) show how many countries in sub-Saharan Africa are likely to achieve ‘OD free status’ by 2015; 3) determine what national level indices influence reductions in OD; and 4) generate hypotheses and recommendations for future studies in this field.

## Methods

To estimate trends of OD prevalence between 2005 and 2010 and to examine how many countries are expected to achieve OD free status by 2015, we selected sub-Saharan African countries satisfying the following three conditions: 1) at least two national level surveys that collected data on OD prevalence between 2000 to 2010; 2) at least one national level survey that collected data on OD prevalence between 2006 and 2010; 3) 10% or more of households reported OD in 2005. Nine countries were dropped from the analysis because of the first two criteria required to get valid estimates of OD prevalence: Burundi, Comoros, Djibouti, Equatorial Guinea, Eritrea, Gabon, Mauritius, Seychelles and Somalia. Following the third criterion that reflects an interest in countries with high levels of OD prevalence (> 10% in 2005), five countries were additionally removed from the analysis: Cameroon, Congo, Gambia, Rwanda and South Africa. The final analysis included 34 countries among 48 sub-Saharan African countries. In addition, because low-income countries may respond differently to national level factors, due to limited resources, we created a sub-population of low-income countries (per capita gross domestic product in 2005 < US$1,000) and examined the association between national level indices and change in OD prevalence among 29 countries.

### Measuring national OD prevalence

This paper estimated OD prevalence in 2005, 2010 and 2015 based on data from national surveys conducted between 2000 and 2010 and reported in the WHO/UNICEF Joint Monitoring Programme for Water Supply and Sanitation country file reports on improved sanitation facilities [14]. A linear trend was used to estimate OD prevalence in 2005, 2010, and 2015 based on the available data points reported by the JMP. National level household surveys included Demographic and Health Surveys, Multiple Indicator Cluster Surveys, World Health Surveys and Malaria Indicator Survey, as well as country census reports. Only national survey data on OD that were validated by the JMP were included in our analysis [see Additional file 1].

### National level indices

Based on recent reports [15,16], we identified three important domains that could influence OD prevalence in sub-Saharan Africa: *Government policy and practice*: (1) implementation of a national sanitation policy; (2) public sector budget line for sanitation, (3) government budget allocation to sanitation; *Economic factors*: (4) per capita gross domestic product (GDP), (5) economic growth, (6) amount of external development assistance for water and sanitation; and *Sanitation approach*: (7) adoption of total sanitation approaches at the national level. We assessed these 7 variables because there is evidence that supports the role of these factors in reducing OD [8,15,16]. In addition, internal conflict was assessed qualitatively to determine its influence on changes in open defecation.

### Government policy and practice

The study assessed whether or not a country has a national sanitation policy and how much national budget was committed to sanitation, based on the 2011 eThekwini Traffic Lights Report – the official mechanism for monitoring sanitation progress and commitments. To characterize the national sanitation policy of each country [17], a dichotomous variable for national sanitation policy was generated: 1) the country has a national sanitation policy that was prepared by government but not yet endorsed by parliament, or the country does not have a sanitation policy; or 2) the country has a sanitation policy that was prepared by government and endorsed by parliament [17].

The study used two different dichotomous variables to assess national budgetary commitment for sanitation. First, a public sector budget line for sanitation was assessed and defined as: 1) the country has a budget allocation for sanitation but it was not used for the sector, or the country has no public budgetary allocation for sanitation; or 2) the country has public sector budget allocations and it was used for the sector. A budget allocated to sanitation was assessed as: 1) the public sector budget allocation to sanitation activities was less than 0.1% of the GDP; or 2) the public sector budgetary allocation to sanitation activities was at least 0.1% to 0.5% of the GDP. This dichotomization was supported by the fact that no country dedicated more than 0.5% of their GDP to sanitation among the 34 countries included in our analysis.

#### Economic factors

Given that the level of national investments in sanitation may be affected by the resources a country has available, or the country’s economic growth [15], we assessed per capita GDP and the GDP growth rate over the five year study period to determine the role of national economic factors on changes in OD prevalence. Data on each country’s per capita GDP (in US$ dollars) were obtained from the World Bank database for 2005 and 2010 [18]. The per capita GDP percent change between 2005 and 2010 was calculated for each country in order to estimate economic growth.

Data on official development assistance for basic drinking water supply and basic sanitation was obtained from the Organization for Economic Co-operation and Development (OECD) database [19]. Per capita aid disbursement (in US$ dollars) was calculated for each country by dividing the average annual aid disbursement for basic drinking water and basic sanitation by the average annual population for the five year study period. Total population estimates were obtained from the WHO/UNICEF database [20].

### Sanitation approach

Data on the integration of community-led total sanitation approaches, or more broadly total sanitation approaches, were included in our analysis. The data were obtained from UNICEF published reports and communicating directly with UNICEF headquarters and regional offices. Countries were classified as having: 1) a national policy/strategy that is under development, so it is unclear whether total sanitation approaches will be part of official policy; 2) total sanitation approaches are implemented but not part of official policy; and 3) total sanitation approaches are explicitly part of the national policy (J. Bevan, personal communication, June 1, 2012).

### Statistical analysis

We applied the Mann–Whitney U test to assess relationships between changes in OD prevalence of sub-Saharan countries between 2005 and 2010 and the following national level factors: 1) national sanitation policy, 2) budget line to sanitation, 3) budget allocated to sanitation, and 4) total sanitation approaches. The Spearman's rank correlation test was used to examine the relationships between changes in OD prevalence and 1) per capita GDP (in US$), 2) GDP growth, and 3) per capita aid disbursement (in US$) for basic drinking water supply and basic sanitation. Data were entered into Excel (Microsoft, USA) and all analyses were performed using STATA/SE 12.0 (Stata Corp, USA).

## Results

### Change in open defecation prevalence

There was wide variability in countries’ progress to reduce OD prevalence, and OD prevalence appeared to increase in several countries. Table 1 presents estimated OD prevalence for 2005, 2010 and 2015, the change in OD prevalence between 2005 and 2010, as well as country-level indices and total sanitation approaches for the 48 sub-Saharan African countries. The study classified countries into four categories based on the change in OD prevalence (Table 1): Group A – countries with large reductions (greater than 10% reduction) in OD prevalence; Group B – countries with mid-level reductions (between 1-9% reduction) in OD prevalence; Group C – countries with no reduction or an increase in OD prevalence (0% to 10% increase); and Group D – countries excluded from study based on our selection criteria (see Methods section). Countries are ordered in each category from the highest to the lowest percent reduction in OD prevalence.

**Table 1:** Summary table of open defecation data for 48 sub-Saharan African countries and national level indices used in the analysis

	**Sub-Saharan Countries**	**OD prevalence**				**National level indices**						
		**2005-2010 Change (%)**	**2005 (%)**^**a**^	**2010 (%)**^**a**^	**Estimated rates for 2015 (%)**^**a**^	**National sanitation policy**^**b**^	**Budget line**^**c**^	**Budget allocated to sanitation**^**d**^	**2005 per capita GDP (US$)**^**e**^	**2005-2010 GDP growth (%)**	**Total sanitation approaches**^**f**^	**2005-2010 annual aid disbursement per capita (US$)**^**g**^
**Group A**	Ethiopia	−22	61	39	18	○	◒	◒	166	116	○	0.3
	Angola	−21	32	12	0	◒	●	●	1,858	140	N/D	0.2
	Sao Tome and Principe	−10	61	51	41	N/D	N/D	N/D	746	60	N/D	2.2
**Group B**	Mozambique	−9	48	39	30	○	◒	●	317	29	○	0.4
	Zambia	−8	21	14	6	◒	○	●	626	100	○	1.0
	Benin	−7	63	56	49	○	◒	●	562	33	●	3.6
	Mali	−5	17	12	7	○	◒	●	403	49	○	1.1
	Guinea	−5	24	19	14	○	◒	●	325	39	○	0.5
	Guinea-Bissau	−5	34	29	24	◒	◒	●	419	38	●	0.3
	Liberia	−5	51	45	40	○	●	●	167	48	○	0.8
	Burkina Faso	−5	64	58	53	○	○	●	382	40	●	1.3
	Uganda	−4	12	8	4	○	○	◒	325	57	N/D	0.6
	Swaziland	−4	19	16	12	◒	○	●	2,540	38	N/D	0.5
	Lesotho	−4	41	38	34	N/D	N/D	N/D	662	52	N/D	1.2
	Togo	−4	53	49	46	◒	◒	●	391	34	○	0.3
	Chad	−4	67	63	59	◒	◒	●	542	25	●	0.7
	Malawi	−3	13	10	7	○	◒	◒	215	58	○	0.2
	Botswana	−3	20	17	15	N/D	N/D	N/D	5,468	35	N/D	0.0
	Ghana	−3	20	18	16	○	○	●	495	168	○	0.7
	Mauritania	−3	51	48	45	◒	○	●	717	46	○	0.8
	Senegal	−2	21	20	18	○	◒	◒	799	29	●	0.9
	Cote d'Ivoire	−2	32	30	28	◒	○	●	908	27	◒	0.1
	Congo, Dem. Rep.	−1	11	11	10	◒	○	●	124	60	N/D	0.1
	Sudan	−1	43	42	42	○	◒	●	713	100	N/D	0.3
	Namibia	−1	55	54	53	N/D	N/D	N/D	3,491	53	N/D	1.4
**Group C**	Central African Republic	0	23	23	23	◒	●	●	336	36	●	0.0
	Kenya	1	14	14	15	○	○	◒	526	51	○	0.6
	Nigeria	1	22	23	24	○	○	●	803	59	○	0.1
	Madagascar	1	41	43	44	○	◒	●	282	49	○	0.1
	Niger	1	80	81	81	○	○	◒	262	37	○	1.0
	Zimbabwe	2	27	29	31	◒	●	●	458	30	N/D	0.4
	Tanzania	3	11	14	18	◒	○	●	373	40	N/D	0.6
	Sierra Leone	5	25	30	35	○	○	◒	240	35	○	0.7
	Cape Verde	10	26	36	45	○	N/D	N/D	2,055	62	●	4.4
**Group D**	Gambia	−3	3	0	0	◒	○	◒	307	52	○	1.6
	South Africa	−3	9	6	3	N/D	N/D	N/D	5,234	39	N/D	0.1
	Burundi	−1	1	0	0	N/D	N/D	N/D	110	75	N/D	0.7
	Gabon	−1	2	1	0	N/D	N/D	N/D	6,322	38	N/D	0.0
	Congo	0	9	8	8	○	○	◒	1,723	72	◒	0.0
	Mauritius	0	0	0	0	N/D	N/D	N/D	5,054	50	N/D	0.0
	Rwanda	0	3	3	3	○	○	◒	281	89	○	0.4
	Comoros	1	1	2	2	●	●	●	602	22	N/D	0.5
	Cameroon	2	7	9	10	N/D	N/D	N/D	945	21	○	0.1
	Somalia	12	56	68	80	N/D	N/D	N/D	277	−58	N/D	0.1
	Djibouti	N/D	N/D	N/D	N/D	◒	◒	◒	877	46	N/D	0.1
	Equatorial Guinea	N/D	N/D	N/D	N/D	◒	○	○	13,521	48	N/D	0.2
	Eritrea	N/D	N/D	N/D	N/D	N/D	N/D	N/D	245	64	N/D	0.4
	Seychelles	N/D	N/D	N/D	N/D	N/D	N/D	N/D	10,661	1	N/D	0.0

Group A: Countries with large reductions (greater than 10% reduction) in open defecation; Group B: Countries with mid-level reduction (between 1-9% reduction) in open defecation; Group C: Countries with no reduction or an increase in open defecation (0% to 10% increase); and Group D: Countries excluded from study based on selection criteria (see Methods section).

^a^ OD prevalence for 2005, 2010 and 2015 were calculated by creating a line equation for each country based on the available and eligible data points between 2000 and 2010 obtained from national surveys that met standards of quality set by Joint Monitoring Programme [14].

^b^ ○ The country has a sanitation policy that is prepared by government and endorsed by parliament; ◒ the country has a national sanitation policy that is prepared by government but not yet endorsed by parliament; or the country does not have a sanitation policy [17].

^c^ ○ The country has public sector budget allocations and it is used for the sector; ◒ the country has budget allocations for sanitation but they are not used for the sector; ● the country has no public budgetary allocation for sanitation [17].

^d^ ○ The public sector budgetary allocation to sanitation activities is at least 0.5% of the GDP; ◒ the public sector budgetary allocation to sanitation activities is between 0.1% to 0.5% of the GDP; ● the public sector budget allocation to sanitation activities is less than 0.1% of the GDP [17].

^e^ Data on each country’s per capita GDP (in US$ dollars) were obtained from the World Bank database for 2005 and 2010 [18].

^f^ ○ Total sanitation approaches are explicitly part of the national policy; ◒ total sanitation approaches are being implemented but not part of official policy; ● a national policy/strategy that is under development, so it is unclear whether total sanitation approaches will be part of official policy (J. Bevan, personal communication, June 1, 2012).

^g^ Per capita aid disbursement (in US$) for basic drinking water and basic sanitation was calculated for each country by dividing the average annual aid disbursement for basic drinking water and basic sanitation by the average annual population for the five year study period [19].

**Figure 1:**
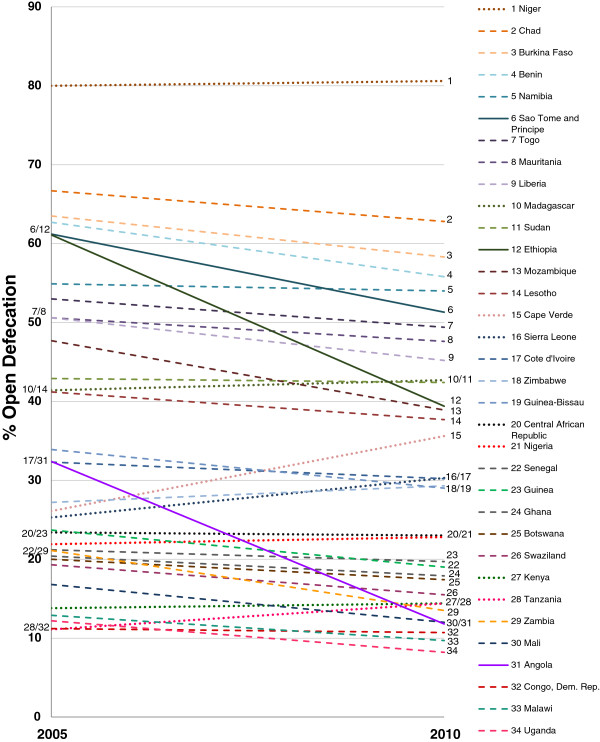
**Change in OD prevalence for 34 sub-Saharan African Countries between 2005–2010.** Data calculated from national surveys for the 34 sub-Saharan African countries that met the following selection criteria: 1) have at least two national surveys that collected data on open defecation conducted between 2000 to 2010, 2) have at least one national survey that collected data on open defecation conducted between 2006 to 2010, and 3) have more than 10% of the households reporting open defecation in 2005 [14]. Solid lines (−) indicate countries with greater than 10% reduction in open defecation; Dashed lines (⋯⋯) indicate countries with 1 to 10% reduction in open defecation rate; Dotted lines (∙-∙∙-∙-) indicates countries with no reduction or increase in OD prevalence.

After excluding the countries that did not meet our selection criteria, 34 countries were further studied to look at changes in OD prevalence (Figure 1). From 2005 to 2010, Ethiopia experienced the highest reduction in its OD prevalence (−22%), followed by Angola (−21%) and Sao Tome and Principe (−10%). OD prevalence in Cape Verde and Sierra Leone, however, increased during this same time period by 10% and 5%, respectively. The majority of the countries included in this study, 22 out of 34, had a mid-level reduction in OD prevalence, between 1% and 9% whereas 9 countries had no reduction or an increase in OD.

**Figure 2:**
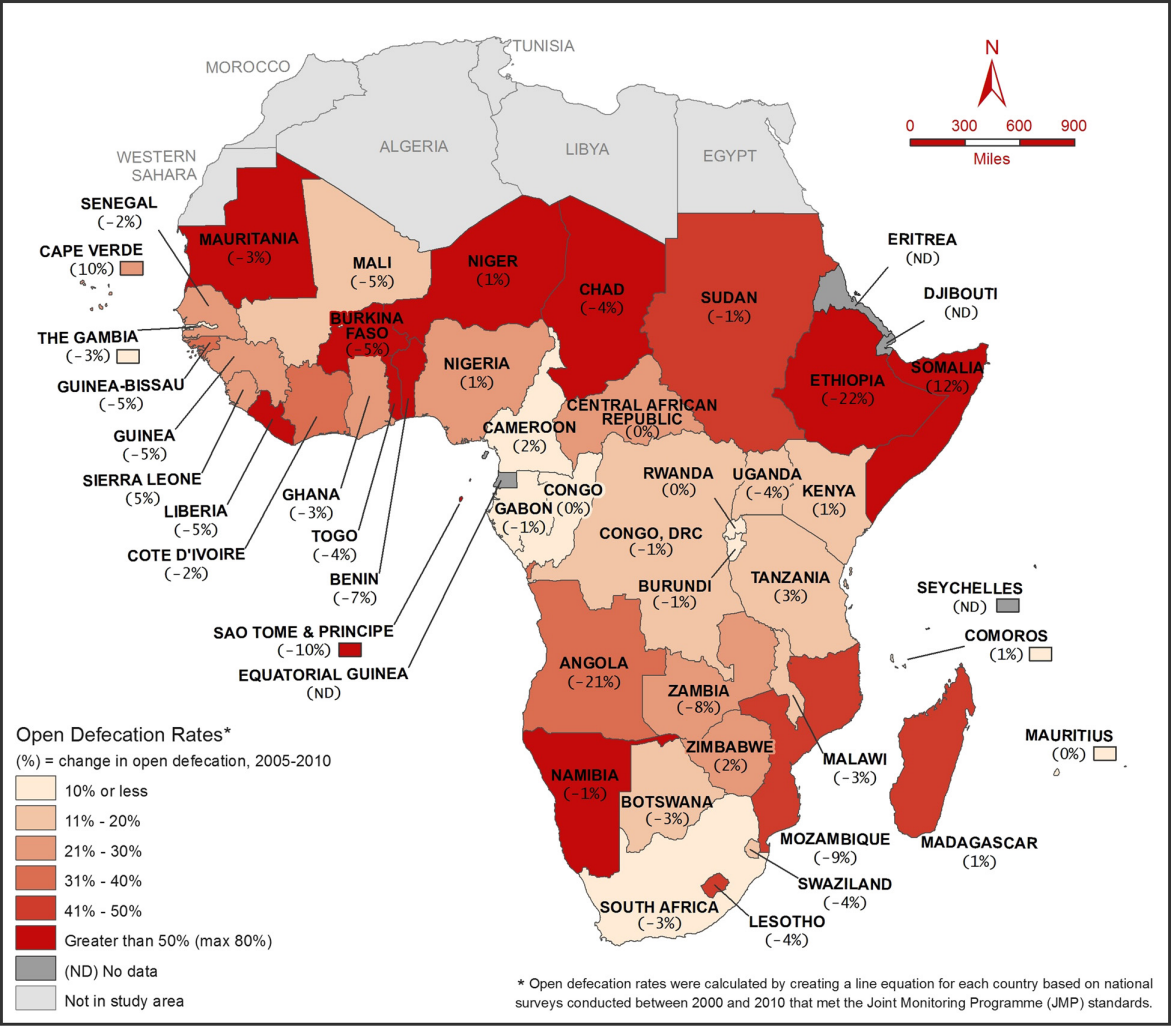
OD prevalence for Sub-Saharan Africa in 2005 (represented by color categories) and Changes in Open Defecation between 2005–2010 (represented by number underneath each country’s name).

Eleven sub-Saharan African countries had greater than 50% OD prevalence in 2005 (Figure 2): Niger (80%), Chad (67%), Burkina Faso (64%), Benin (63%), Ethiopia (61%), Sao Tome and Principe (61%), Somalia (56%), Namibia (55%), Togo (53%), Liberia (51%) and Mauritania (51%). Niger, which had the highest prevalence in 2005, increased OD by 1% from 2005 to 2010. Among the 11 countries with high OD (>50%) in 2005, only two countries (i.e. Ethiopia and Sao Tome and Principe) achieved more than 10% reduction in OD prevalence between 2005 and 2010. Table 1 presents estimated OD prevalence for 2015. Based on the trend (2000–2010), 6 out of 34 countries are expected to reach equal to or less than 10% OD by 2015 and only one country, Angola, may achieve ‘OD free status’ by 2015: Angola (0%), Uganda (4%), Zambia (6%), Malawi (7%), Mali (7%) and Democratic Republic of the Congo (10%). In addition, 4 countries may continue to have greater than 50% OD: Burkina Faso (53%), Chad (59%), Namibia (53%) and Niger (81%).

### National level indices and open defecation

No association was observed between all of seven national indices and reduction in OD prevalence between 2005 and 2010. In a subset analysis using 29 low-income countries (GDP in 2005 < US$ 1000), per capita aid disbursement (in US$) for basic drinking water supply and basic sanitation (hereafter, per capita aid disbursement for WSS) was significantly associated with reduction in OD prevalence between 2005 and 2010 whereas other six indices did not show significant association.

### Government policy and practice and open defecation

Eighteen among 34 countries had a national sanitation policy that was prepared by government and endorsed by parliament. The public sector budget allocated and used for sanitation was observed in 13 countries; however none of the countries allocated more than 0.5% of the GDP to the sanitation sector. There was no association observed between 2005–2010 in the reduction in OD prevalence and the presence of a national sanitation policy, a public sector budget line or budget allocations. Angola, as an example, had a 21% reduction in OD, but has no national sanitation policy endorsed by parliament, no public sector budget line and invests less than 0.1% of its GDP in sanitation. In contrast, Kenya, Niger and Sierra Leone showed very little progress towards reducing OD, though each has a national sanitation policy endorsed by parliament, a public sector budget line and investment of between 0.1 - 0.5% of GDP in sanitation.

### Economic factors and open defecation

In 2005, per capita GDP ranged from US$ 124 in Democratic Republic of the Congo to US$ 5,468 in Botswana. Chad had the lowest GDP growth for the period 2005–2010 at 25%, compared to Ghana which had the highest (168%). Per capita GDP and economic growth were not associated with changes in OD prevalence. Ethiopia had the second lowest per capita GDP in 2005 (US$ 166) and exhibited the highest OD reduction (22%). Cape Verde, which had the fourth highest per capita GDP in 2005 (US$ 2,055) had, in contrast, a 10% increase in OD prevalence from 2005 to 2010. Regarding economic growth and OD reduction, Mozambique had the third lowest GDP growth between 2005 and 2010 (29%) but managed to decrease OD prevalence by 9% during that time period. Ghana, in contrast, was the country with the highest GDP growth, and presented a mid-level reduction in OD of 3%. This suggests that per capita GDP and economic growth are not related to reductions in OD prevalence for these 34 sub-Saharan African countries between 2005 and 2010.

In order to investigate the relationship between external development assistance and reduction in OD prevalence, annual per capita aid disbursement for WSS was calculated for each country for the period from 2005 to 2010. A total of 8 countries had an annual per capita aid disbursement for WSS greater than US$ 1: Zambia (US$ 1.00), Mali (US$ 1.10), Lesotho (US$ 1.20), Burkina Faso (US$ 1.30), Namibia (US$ 1.40), Sao Tome and Principe (US$ 2.20), Benin (US$ 3.60) and Cape Verde (US$ 4.40). There was no observed relationship between aid disbursement and OD reduction when the analysis was conducted for all 34 sub-Saharan African countries. However, when we analyzed the effect of aid disbursements for 29 low-income countries (countries with per capita GDP in 2005 greater than US$1,000 were excluded: Angola, US$ 1,858; Cape Verde, US$ 2,055; Swaziland, US$ 2,540; Namibia, US$ 3,491; and Botswana, US$ 5,468), a positive and significant association was observed between reduction in OD prevalence and per capita aid disbursement for WSS (p-value = 0.02).

### Sanitation approach and open defecation

Total sanitation approaches, which includes community-led total sanitation and community approaches to total sanitation, were introduced to sub-Saharan African countries in 2006 [10]. Since that time, many countries have adopted this approach explicitly as part of the national policy – that was observed in 15 out of 23 countries where data were available: Ethiopia, Ghana, Guinea, Kenya, Liberia, Madagascar, Malawi, Mali, Mauritania, Mozambique, Niger, Nigeria, Sierra Leone, Togo and Zambia. Based on personal communication with UNICEF (J. Bevan, personal communication, June 1, 2012) (T. Dooley, personal communication, June 11, 2012), these countries are in the process of scaling up the application of total sanitation approaches as a means of reducing OD prevalence. Benin, Burkina Faso, Cape Verde, Central African Republic, Chad, Guinea-Bissau and Senegal are each in the process of developing a national sanitation policy and strategy, and it’s unclear whether total sanitation approaches will be incorporated. At this point, no association was observed between a reduction in OD prevalence and the presence of total sanitation approaches as part of the countries’ national policy.

## Discussion

Our findings suggest that only a limited number of countries in sub-Saharan Africa have made significant progress toward reducing OD prevalence and only one country among 34 countries analyzed, Angola, is expected to end OD by 2015. In the subset analysis after excluding the countries with per capita GDP > $1,000, a higher level of per capita aid disbursement for WSS was positively associated with a reduction in OD prevalence. To our knowledge, this is one of the first studies to explore trends in OD prevalence over the past decade and to examine national level indices, which could influence reduction in OD prevalence among sub-Saharan African countries.

Several studies have highlighted the importance of developing national sanitation policies to provide the necessary framework in which sanitation initiatives and targets are combined into a single program at the national level [21]. Currently, our study found that 18 out of 34 sub-Saharan African countries had a national sanitation policy that was prepared by government and endorsed by parliament. Thirty-two sub-Saharan African countries, however, signed a declaration in 2008 to have a national sanitation policy as a part of the eThekwini Declaration [22].

Extending and sustaining sanitation programs and implementing sanitation policies require adequate budget allocations and management. In our study, only 7 out of 34 countries allocated between 0.1% - 0.5% of the GDP to sanitation. None of 34 countries had a sanitation budget equal to or more than 0.5% of GDP, which was a mandate of the eThekwini declaration commitments. This allocation of resources does not appear sufficient to improve sanitation in line with the sanitation target of the MDGs. A World Bank report estimates that for sub-Saharan Africa, approximately 0.9% of the region’s GDP must be spent annually to meet the sanitation MDG target, which aims to halve the proportion of people without access to improved sanitation [15]. Furthermore, it is estimated that for most countries, the major source of investment in sanitation and drinking water comes from central governments, not donors [8].

The ability of a country to improve sanitation conditions is strongly correlated to its economic condition [15]. Our study did not find a significant association between economic conditions (i.e. per capita GDP and GDP growth) and reduction in OD prevalence. This finding should be interpreted carefully. First, it may be that countries are not investing in sanitation or are investing at the high end of the sanitation ladder, such as piped sewerage systems, instead of improving basic sanitation, which includes OD. Second, although we did not find a significant association, among the three countries which reduced the prevalence of OD between 2005 and 2010 by 10% or more, two countries (i.e. Ethiopia and Angola) showed the highest GDP growth during the same period (Table 1), implying that economic growth may influence reductions in OD prevalence. Finally, it may be that countries are spending more on water supply rather than sanitation – the 2010 GLASS report states that only one fifth of the financing devoted to sanitation and drinking-water combined is spent on sanitation [8].

Our analysis found per capita aid disbursements for basic WSS to be associated with a reduction in OD prevalence in low-income countries of sub-Saharan Africa. Development assistance continues to be an important source of financing for sanitation and drinking-water supply programs, especially in low-income countries. In 2010, sub-Saharan Africa received, in absolute terms, larger amount of aid for sanitation and drinking-water than any other region in the world [8]. It is important to note that per capita aid disbursement was calculated using amounts allocated for both basic drinking water supply and basic sanitation. At this point, the data were not available to calculate per capita aid disbursement allocated exclusively to sanitation.

A total of 15 countries included in our analysis have total sanitation approaches explicitly as part of their national policy. Total sanitation approaches appear to be an innovative method for empowering communities to achieve ‘OD free status’. It focuses on raising awareness of the risks associated with OD in order to stimulate a collective change in sanitation behavior among communities where OD is commonly practiced. It promotes the use of local sustainable and affordable sanitation options, rather than focusing on hardware subsides [10-12]. Total sanitation approaches were introduced in sub-Saharan Africa in 2006 and have been progressing at a variable pace since that time [10]. Given its limited time in sub-Saharan Africa, very little can be said about the relationship between total sanitation approaches and reductions in OD prevalence. Many studies have, however, shown the success of total sanitation approaches in achieving OD free villages [10-13,23]. In Ethiopia, over 15,000 have reached OD free status [24]. The government of Zambia has reported that this approach has resulted in more than 210,000 people living in OD free communities [10]. In addition, researchers have found that total sanitation approaches in Zambia have substantially increased sanitation access and use in just one year – far surpassing the success rate of subsidized programs of the past [13].

We did not systemically assess internal conflict, which can detrimentally affect development programs of all kinds, including sanitation programs [25]. However, this paper found, among 9 countries that showed no reduction, or showed an increase in OD prevalence, 6 of them are considered fragile states: Central Africa Republic, Kenya, Nigeria, Niger, Sierra Leone and Zimbabwe based on OECD data, implying the impact of internal conflict on reducing OD [26]. Although this was not included in our analysis, internal conflict and its impact in reducing open defecation should be explored further on future studies.

This study has a number of limitations. First and foremost, we used national level factors that may have a significant time lag between when they were implemented and our outcome measure. Thus, more time may be needed to observe the changes resulting from these national level factors. Second, we used different numbers of national surveys to estimate OD prevalence for each country. Some countries have had several surveys in the past decade, while others had only two surveys conducted between 2000 and 2010. Applying a line equation to estimate OD prevalence from 2005 and 2010 for those countries with a small number of data points may not represent the actual change in OD prevalence in the country. Third, there were differences between rural and urban OD prevalence that were not addressed in this study. Our study focused on total/national OD prevalence, but some countries may have decreased urban OD prevalence while increasing rural, or vice-versa. Fourth, this study used a limited number of national level variables that are thought to influence OD prevalence. It is possible that we missed relevant information, such as the amount of human resources dedicated to reducing OD prevalence, as well as cultural and behavioral practices related to OD. In addition, national level indices were used as a proxy for measuring a country’s commitment and investment in sanitation. For example, having a national sanitation policy does not necessarily equate to actions being taken to improve sanitation; and, having a budget allocated to sanitation does not always indicate financial investments in reducing OD prevalence. Furthermore, information regarding the amount of external development assistance was obtained for basic drinking water supply and basic sanitation; at this point, per capita aid disbursement for sanitation could not be determined for the studied time period.

One of the goals of this study was to evaluate the association between total sanitation approaches and reduction of OD prevalence. There were several obstacles to exploring the scale of total sanitation approaches used in sub-Saharan Africa and its impact in OD prevalence. First, total sanitation approaches may still be too early in development and the magnitude of implementation is very different across countries. Even though it may be part of a country’s national policy, it does not necessary mean it has been taken to scale. Second, there is no literature on the latency period of the impact of total sanitation approaches on OD prevalence. Lastly, total sanitation approaches are mainly present in rural areas, and our study looked at OD prevalence at the national level including both of urban and rural OD.

One of the key strengths of this study is that there have been limited analyses that calculate changes in OD prevalence and statistically analyze national level indices that may influence changes in sub-Saharan African countries. Several papers have suggested the importance of having a national sanitation policy and a budget allocated to sanitation, but little research has been done to explore the relationship between these national level factors together quantitatively. In addition, this analysis is the first to estimate how many countries will achieve OD free status by 2015, which will likely be useful for developing new development goals related to reducing OD prevalence. Lastly, we calculated the OD prevalence for 2005 and 2010 based on national surveys validated by JMP standards, and assessed which countries had the highest and lowest annual percent change in OD prevalence during this time frame.

## Conclusions

In Africa, diarrheal disease is one of the leading causes of mortality for children under 5 [27]. Studies have shown that interventions to improve human excreta disposal facilities are effective in preventing diarrheal diseases [2]. A very limited number of countries are making significant strides to reduce OD prevalence, which is a fundamental step towards reaching improved sanitation, a target of the MDGs, and reducing deaths from diarrheal diseases. Based on our exploratory analysis, national sanitation policy, budget line and budget allocated to sanitation did not appear to be associated with reductions in OD prevalence. Further, higher per capita GDP and economic growth showed no relationship with the reduction in OD prevalence. Per capita aid disbursement for WSS, however, had a strong relationship to OD reduction in low-income countries. Internal conflict emerged as a potential obstacle for sanitation progress. Lastly, 2015 OD prevalence was estimated, and the results suggest that the goal of ending OD practices in sub-Saharan Africa by 2015 is unlikely to be achieved based on current trends, except in one country, Angola, among 34 countries in our analysis.

There is a need to better understand the role of total sanitation approaches and to determine ways to measure its impact in reducing OD prevalence in sub-Saharan Africa. Based on the current state of OD prevalence in a number of sub-Saharan African countries, and with the 2015 MDGs rapidly approaching, a clearer understanding of the key driving factors for reducing OD prevalence are needed as well as a stronger focus on providing this most basic foundation for health and development.

## Abbreviations

WSS: Basic drinking water supply and basic sanitation; GLASS: Global analysis and assessment of sanitation and drinking-water; GDP: Gross domestic product; JMP: Joint monitoring programme; MDG: Millennium development goal; OD: Open defecation; OECD: Organization for Economic Co-operation and Development; UNICEF: United Nations Children's Fund; WHO: World Health Organization.

## Competing interests

The authors declare that they have no competing interests.

## Authors’ contributions

JPG conceived the concept of this paper. DIG collected the data and drafted the first manuscript. DIG, JPG, and SSK developed the study design, performed the statistical analyses and revised the manuscript. All authors read and approved the final manuscript.

## Pre-publication history

The pre-publication history for this paper can be accessed here:

http://www.biomedcentral.com/1471-2458/13/527/prepub

## Supplementary Material

Additional file 1: Table S1 Surveys used to calculate open defecation prevalence for 34 sub-Saharan African countries. Click here for file

## References

[B1] WHO The global burden of disease: 2004 update 2008 Geneva: World Health Organization

[B2] Clasen TF Bostoen K Schmidt W-P Boisson S Fung IC-H Jenkins MW Scott B Sugden S Cairncross S Interventions to improve disposal of human excreta for preventing diarrhoea Cochrane Database Syst Rev 2010 6 10.1002/14651858.CD007180.pub2 PMC6532559 20556776

[B3] Aziz KMA Hoque BA Hasan KZ Patwary MY Huttly SRA Rahaman MM Feachem RG Reduction in diarrhoeal diseases in children in rural Bangladesh by environmental and behavioural modifications Trans R Soc Trop Med Hyg 1990 84 433 438 10.1016/0035-9203(90)90353-G 2260182

[B4] Garrett V Ogutu P Mabonga P Ombeki S Mwaki A Aluoch G Phelan M Quick RE Diarrhoea prevention in a high-risk rural Kenyan population through point-of-use chlorination, safe water storage, sanitation, and rainwater harvesting Epidemiol Infect 2008 136 1463 1471 10.1017/S095026880700026X 18205977 PMC2870746

[B5] WHO The World health report: 2005: make every mother and child count 2005 Geneva: World Health Organization 10.1080/14034940500217037 16332605

[B6] UNICEF Pneumonia and diarrhoea - Tackling the deadliest diseases for the world’s poorest children 2012 New York: United Nations Children’s Fund

[B7] WHO/UNICEF Progress on Drinking Water and Sanitation Report 2012 2012 New York: WHO/UNICEF Joint Monitoring Programme for Water Supply and Sanitation (JMP)

[B8] WHO UN-Water Global Analysis and Assessment of Sanitation and Drinking-Water (GLASS) 2012 report: the challenge of extending and sustaining services 2012 Geneva: World Health Organization

[B9] Jenkins MW Curtis V Achieving the 'good life': why some people want latrines in rural Benin Soc Sci Med 2005 61 2446 2459 10.1016/j.socscimed.2005.04.036 15949883

[B10] Kar K Milward K Digging in, Spreading out and Growing up: Introducing CLTS in Africa IDS Practice Papers 2011 2011 01 64

[B11] Chambers R Going to Scale with Community-Led Total Sanitation: Reflections on Experience, Issues and Ways Forward IDS Practice Papers 2009 2009 01 50

[B12] Hickling S Bevan J Scaling up CLTS in sub-Saharan Africa Participatory Learning & Action (PLA) 61 – Tales of Shit: Community-Led Total Sanitation in Africa 2010 London: International Institute for Environment and Development (IIED)

[B13] Harvey PA Zero subsidy strategies for accelerating access to rural water and sanitation services Water Sci Technol 2011 63 1037 1043 21411956 10.2166/wst.2011.287

[B14] WHO/UNICEF Estimates for the Use of Improved Sanitation Facilities. Joint Monitoring Programme for Water Supply and Sanitation (JMP) 2012 http://www.wssinfo.org/documents-links/documents/?tx_displaycontroller[type]=country_files, July 23, 2012

[B15] Morella E Foster V Ghosh Banerjee S Climbing the Ladder: The State of Sanitation in Sub-Saharan Africa 2008 Washington, DC: The World Bank

[B16] Perez E Cardosi J Coombes Y Devine J Grossman A Kullmann C Kumar CA Mukherjee N Prakash M Robiarto A What Does It Take to Scale Up Rural Sanitation 2012 Washington, D.C: The World Bank, Water and Sanitation Program

[B17] AMCOW, WSP, UNICEF, WaterAid, CREPA Sanitation and Hygiene in Africa at a Glance - A synthesis of country priority actions 2011 Abuja, Nigeria: African Minister's Council on Water

[B18] World Bank Database - GDP per capita http://data.worldbank.org/indicator/NY.GDP.PCAP.CD, 23 July, 2012

[B19] Official Development Assitance Organization for Economic Co-operation and Development (OECD) 2012 http://stats.oecd.org/Index.aspx?DataSetCode=CRS1, July 23, 2012

[B20] WHO/UNICEF Population Estimates. Joint Monitoring Programme for Water Supply and Sanitation (JMP) 2012 http://www.wssinfo.org/data-estimates/table/, July 23, 2012

[B21] Tayler K Scott R Sanitation policy: making it work Waterlines 2006 25 25 26

[B22] WSP The eThekwini Declaration and AfricaSan Action Plan 2008 Africa: Water and Sanitation Program

[B23] Kalimuthu A Hossain Y Crossfire: 'Community-led total sanitation is the best method of achieving sustainable sanitation for all in rural areas' Waterlines 2008 27 177 183 10.3362/1756-3488.2008.022

[B24] Community-Led Total Sanitation Ethiopiahttp://www.communityledtotalsanitation.org/country/ethiopia, 23 July 2012

[B25] World B World Development Report 2011: Conflict, security and development 2011 Washington, DC: The World Bank

[B26] OECD-DAC Ensuring Fragile States Are Not Left Behind - 2011 Factsheet on Resource Flows in Fragile States 2011 Paris, France: OECD-DAC International Network on Conflict and Fragility

[B27] Liu L Johnson HL Cousens S Perin J Scott S Lawn JE Rudan I Campbell H Cibulskis R Li M Global, regional, and national causes of child mortality: an updated systematic analysis for 2010 with time trends since 2000 Lancet 379 2151 2161 22579125 10.1016/S0140-6736(12)60560-1

