# Supplementary material for: Exploring changes in open defecation prevalence in sub-Saharan Africa based on national level indices
Source: BMC Public Health. 2013 May 30;13:527. doi: 10.1186/1471-2458-13-527 (PMC3679748; doi:10.1186/1471-2458-13-527)
Supplement: Additional file 1: Table S1 — Surveys used to calculate open defecation prevalence for 34 sub-Saharan African countries. [file 1471-2458-13-527-S1.docx]

**Supplemental Material**

**Exploring Changes in Open Defecation Prevalence in sub-Saharan Africa based on National Level Indices**

Deise I. Galan, Seung-Sup Kim and Jay P. Graham

**Surveys used to calculate open defecation prevalence for 34 sub-Saharan African countries**

Open defecation (OD) prevalence was estimated for 2005, 2010 and 2015 based on data from national surveys conducted between 2000 and 2010 and reported in the WHO/UNICEF Joint Monitoring Programme for Water Supply and Sanitation country file reports on improved sanitation facilities [1]. A linear trend was used to estimate OD prevalence in 2005, 2010, and 2015 based on the available data points reported by the JMP. National level household surveys include Demographic and Health Surveys, Multiple Indicator Cluster Surveys, World Health Surveys and Malaria Indicator Survey, as well as country census reports. Only national survey data on OD that were validated by the JMP were included in our analysis. Table S1 indicates which surveys were used for each of the 34 sub-Saharan African countries analyzed in our paper. JMP country file reports can be obtained for each country through the following link:

[http://www.wssinfo.org/documents-links/documents/?tx_displaycontroller[type]=country_files](http://www.wssinfo.org/documents-links/documents/?tx_displaycontroller%5btype%5d=country_files).

**Table S1. Surveys used to calculate open defecation prevalence for 34 sub-Saharan African countries**

| **Sub-Saharan Countries** | **Surveys used to calculate OD rates between 2000-2010** |
| --- | --- |
| Angola | MICS 2001; MIS 2007 |
| Benin | DHS 2001; CEN 2002; QUIBB 2003; ESAFEM 2003; DHS 2006; AGVSAN 2009 |
| Botswana | MICS 2000; CEN 2001; BDS 2006; BFH 2007 |
| Burkina Faso | QUIBB 2003; EBCVM 2003; DHS 2003; QUIBB 2005; CEN 2006; MICS 2006; QUIBB 2007; EPM 2009; ENA 2010 |
| Cape Verde | IDRF 2002; QUIBB 2007; CEN 2010 |
| Central African Republic | MICS 2000; MICS 2006 |
| Chad | MICS 2000; WHS 2003; ECSI 2003; DHS 2004; MICS 2010 |
| Congo, Dem. Rep. | MICS 2001; ENM 2005; ENQ 2005; DHS 2007 |
| Cote d'Ivoire | MICS 2000; ENVM 2002; AIS 2005; MICS 2006; ENV 2008 |
| Ethiopia | DHS 2000; WMS 2000; WHS 2003; WMS 2004; DHS 2005; DHS 2010 |
| Ghana | CEN 2000; DHS 2003; WHS 2003; CWIQ 2003; LSS 2006; MICS 2006; DHS 2008; SAGE 2008 |
| Guinea | QUIBB 2002; MICS 2003; DHS 2005; ENS 2007; ELEP 2007 |
| Guinea-Bissau | QUIBB 2002; MICS 2006 |
| Kenya | MICS 2000; DHS 2003; WHS 2004; IHBS 2006; DHS 2009; CEN 2009 |
| Lesotho | MICS 2000; HIES 2002; DHS 2004; CEN 2006; DHS 2009 |
| Liberia | DHS 2000; DHS 2007; CWIQ 2007; CEN 2008; LMIS 2009 |
| Madagascar | MICS 2000; EP 2002; EP 2004; DHS 2004; EP 2005; DHS 2009; EPM 2010 |
| Malawi | DHS 2000; CWIQ 2002; WHS 2003; DHS 2004; MICS 2006; CEN 2008; DHS 2010 |
| Mali | EMEP 2001; DHS 2001; ELIM 2003; DHS 2006; ELIM 2006; MICS 2010 |
| Mauritania | EPCV 2000; DHS 2001; EMIP 2004; EPCV 2004; MICS 2007 |
| Mozambique | QUIBB 2001; CWIQ 2002; HBS 2003; DHS 2003; IFTRAB 2005; CEN 2007; MICS 2008 |
| Namibia | DHS 2000; CEN 2001; WHS 2003; DHS 2007 |
| Niger | MICS 2000; QUIBB 2005; DHS 2006 |
| Nigeria | CLS 2000; DHS 2003; NLSS 2004; CWIQ 2006; GHS 2006; MICS 2007; BLS 2008; DHS 2008 |
| Sao Tome and Principe | MICS 2000; CVF 2000; MICS 2006 |
| Senegal | MICS 2000; ESAM 2002; WHS 2003; DHS 2005; MIS 2006; MIS 2009 |
| Sierra Leone | MICS 2000; IHS 2003; CEN 2004; MICS 2006; DHS 2008 |
| Sudan | SHHS 2006; CEN 2008 |
| Swaziland | HIES 2000; MICS 2000; WHS 2003; DHS 2007; pMICS 2010 |
| Tanzania | HSB 2000; HBS 2001; CEN 2002; AIS 2003; DHS 2005; HBS 2007; AIS 2008; DHS 2010 |
| Togo | MICS 2000; MICS 2006; QUIBB 2006; MICS 2010 |
| Uganda | NHS 2000; DHS 2001; CEN 2002; NHS 2002; NHS 2003; DHS 2005; NHS 2006; DHS 2006; NHS 2009; MIS 2009 |
| Zambia | CEN 2000; LCMS 2002; WHS 2003; LCMS 2004; LCMS 2006; DHS 2007 |
| Zimbabwe | WHS 2003; DHS 2005; NSS 2008; MIMS 2009 |

**Abbreviations**

| AGVSAN | Analyse Globale de la Vulnérabilité, de la Sécurité Alimentaire et de la Nutrition -Benin |
| --- | --- |
| AIS | HIV/Aids Indicator Survey |
| BDS | Botswana Demographic Survey, |
| BFH | Botswana Family Health Survey |
| BLS | Nigeria Water Supply and Sanitation Baseline Survey |
| CEN | National Census |
| CLS | Child Labor Survey |
| CVF | Inquérito sobre Condições de Vida das Familias - Sao Tome and Principe |
| CWIQ | Core Welfare Indicator Questionnaire |
| DHS | Demographic and Health Survey |
| EBCVM | Enquête burkinabé sur les conditions de vie des ménages - Burkina Faso |
| ECSI | Enquête sur la Consommation et le Secteur Informel - Chad |
| ELEP | Enquête Légère pour l'Evaluation de la Pauvreté - Guinea |
| ELIM | Enquête Légère Intégrée auprès des Ménages - Mali |
| EMEP | Enquête Malienne sur l'Evaluation de la Pauvreté - Mali |
| EMIP | Enquête sur la Mortalité Infantile et le Paludisme -Mauritania |
| ENA | Enquête Nationale Sur L’Accès des Ménages - Burkina Faso |
| ENM | Enquête Nationale auprès des Ménages - Dem. Rep. Congo |
| ENQ | Enquête 1-2-3 sur l'emploi, le secteur informel et les conditions de vie des ménages - Dem. Rep. Congo |
| ENS | Enquête Nationale Sur L'état Nutritionnel et le suivi des principaux indicateur de suivi de - Guinea |
| ENV | Enquête Niveau de Vie des Ménages - Cote d'Ivoire |
| ENVM | Enquête Niveau de Vie des Ménages - Cote d'Ivoire |
| EP | Enquête Prioritaire |
| EPCV | Enquête permanente sur les conditions de vie des ménages - Mauritania |
| EPM | Enquêtes Périodiques auprès des Ménages |
| ESAFEM | Enquête Socio- Anthropologique Sur la Fécondité et la Mortalité - Benin |
| ESAM | Enquête Sénégalaise Auprès des Ménages - Senegal |
| GHS | General Household Survey |
| HBS | Household Budget Survey |
| HIES | Household Income and Expenditure Surveys |
| HSB | Household Budget Survey - Tanzania |
| IDRF | Inquérito às Despesas e Receitas Familiares - Cape Verde |
| IFTRAB | Inquérito Integrado à Força de Trabalho - Mozambique |
| IHBS | Kenya Integrated Household Budget Survey |
| IHS | Integrated Household Survey - Sierra Leone |
| LCMS | Living Conditions Monitoring Survey |
| LSS | Living standards Survey |
| MICS | Multiple Indicator Cluster Survey |
| MIMS | Multiple Indicator Monitoring Survey |
| MIS | Malaria Indicator Survey |
| NHS | National Household Survey |
| NLSS | Nigerian Living Standard Survey - Nigeria |
| NSS | Nutrition Surveillance Survey |
| QUIBB | Basic Well-Being Indicator Questionnaire |
| SAGE | Study on Global Ageing and Adult Health |
| SHHS | Sudan Household Health Survey |
| WHS | World Health Survey |
| WMS | Welfare Monitoring Survey |

**Supplemental Material Reference**

1. WHO/UNICEF: *Estimates for the Use of Improved Sanitation Facilities.* Joint Monitoring Programme for Water Supply and Sanitation (JMP); 2012 [<http://www.wssinfo.org/documents-links/documents/>, July 23, 2012].
